# A Bipolar Membrane Containing Core–Shell Structured Fe_3_O_4_-Chitosan Nanoparticles for Direct Seawater Electrolysis

**DOI:** 10.3390/membranes16010023

**Published:** 2026-01-02

**Authors:** Hyeon-Bee Song, Eun-Hye Jang, Moon-Sung Kang

**Affiliations:** Department of Green Chemical Engineering, College of Engineering, Sangmyung University, Cheonan 31066, Republic of Korea; 2020D3008@sangmyung.kr (H.-B.S.); 2014D8006@sangmyung.kr (E.-H.J.)

**Keywords:** hydrogen production, direct seawater electrolysis, inorganic precipitates, bipolar membrane, water-splitting performance, chitosan, iron oxide, core–shell catalyst

## Abstract

Seawater has attracted increasing attention as a promising resource for hydrogen production via electrolysis. However, multivalent ions present in seawater can reduce the efficiency of direct seawater electrolysis (DSWE) by forming inorganic precipitates at the cathode. Bipolar membranes (BPMs) can mitigate precipitate formation by regulating local pH, thereby enhancing DSWE efficiency. Accordingly, this study focuses on the fabrication of a high-performance BPM for DSWE applications. The water-splitting performance of BPMs is strongly dependent on the properties of the catalyst at the bipolar junction. Herein, iron oxide (Fe_3_O_4_) nanoparticles were coated with cross-linked chitosan to improve solvent dispersibility and catalytic activity. The resulting core–shell catalyst exhibited excellent dispersibility, facilitating uniform incorporation into the BPM. Water-splitting flux measurements identified an optimal catalyst loading of approximately 3 μg cm^−2^. The BPM containing Fe_3_O_4_–chitosan nanoparticles achieved a water-splitting flux of 26.2 μmol cm^−2^ min^−1^, which is 18.6% higher than that of a commercial BPM (BP-1E, Astom Corp., Tokyo, Japan). DSWE tests using artificial seawater as the catholyte and NaOH as the anolyte demonstrated lower cell voltage and stable catholyte acidification over 100 h compared to the commercial membrane.

## 1. Introduction

Hydrogen is widely recognized as a key energy carrier for the development and maintenance of a low-carbon economy [1]. Recently, as the cost of electricity from renewable energy sources such as solar and wind power continues to decrease, green hydrogen production through water electrolysis has been gaining increasing attention. Water electrolysis generally requires highly purified water. Proton-exchange membrane water electrolysis (PEMWE) uses ultrapure water [2], whereas alkaline electrolysis employs a 20–30% potassium hydroxide (KOH) aqueous solution [3]. Meanwhile, producing high-purity water requires costly purification and desalination processes, which involve substantial expenses for equipment, land, maintenance, and transportation. To address these challenges, the direct use of seawater, which accounts for approximately 96.5% of the Earth’s water resources, for water electrolysis has gained increasing attention.

Seawater electrolysis is generally classified into indirect seawater electrolysis (ISWE) and direct seawater electrolysis (DSWE). The primary distinction between these two approaches lies in the requirement for a pretreatment process. ISWE involves converting seawater into high-purity water through desalination processes, such as reverse osmosis or electrodialysis, followed by electrolysis. This additional pretreatment step leads to increased costs and energy losses, which can account for up to 7% of the total system cost [4,5]. In contrast, DSWE directly utilizes seawater and can be integrated with fuel cell systems. This approach has the potential to not only store surplus renewable energy and generate electricity, but also to simultaneously supply clean drinking water and renewable energy in arid regions [6]. Typically, DSWE employs an alkaline electrolyte, such as 1 M KOH, to suppress the chlorine evolution reaction (ClER) and promote the oxygen evolution reaction (OER). However, this process generates alkaline wastewater containing unreacted bases and residual ions, such as Cl^−^, Mg^2+^, and Ca^2+^, which complicates wastewater management and discharge [7]. In addition, seawater contains various multivalent ions, and their reactions during electrolysis lead to the formation of inorganic precipitates, particularly magnesium hydroxide (Mg(OH)_2_), as the pH near the cathode increases. These precipitates can block the active sites of the electrode catalyst and significantly increase the cell voltage.

**Figure 1:**
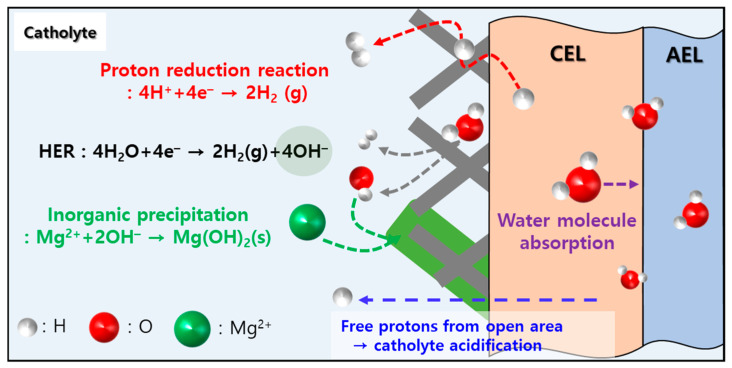
Schematic diagram illustrating the mechanism of catholyte acidification during direct seawater electrolysis (Red dashed line: reduction reaction driven by H^+^ generated in BPM; gray dashed line: H^+^ production reaction from water molecules; green dashed line: formation of inorganic precipitates by multivalent ions present in seawater).

To overcome these issues, a bipolar membrane (BPM) can be applied to seawater electrolysis [8,9]. Figure 1 illustrates the acidification mechanism of the catholyte through a BPM in the DSWE process. The H^+^ ions generated by the BPM neutralize the OH^−^ ions produced after the hydrogen evolution reaction (HER) at the cathode, which prevents the formation of inorganic precipitates and maintains the solution at a low pH (e.g., below 3). In addition, the H^+^ ions generated through water splitting in the BPM contribute to increased hydrogen production through a reduction reaction. For these reasons, the use of a BPM can overcome the limitations of DSWE technology and significantly improve overall process performance [10].

A BPM is a unique ion-exchange membrane (IEM) composed of an anion-exchange layer (AEL) and a cation-exchange layer (CEL) joined together. When a voltage is applied under reverse-bias conditions, water molecules dissociate at the bipolar junction, and the resulting hydrogen ions (H^+^) and hydroxide ions (OH^−^) migrate through the CEL and AEL, respectively [11]. The rate of water-splitting depends strongly on the characteristics of the bipolar junction [12]. To enhance the water-splitting performance of BPMs, a thin layer of water-splitting catalyst is typically introduced between the CEL and AEL. Examples of such catalysts include carboxylic acids, weak bases, and inorganic materials such as metal oxides or hydroxides [13]. In particular, a thin layer of metal oxide or hydroxide can reduce the water-splitting potential by increasing the hydrophilicity of the interface, thereby improving the mobility of H^+^ and OH^−^ ions [14]. Furthermore, Hanada et al. reported that metal ions in the bipolar junction interact with water molecules, loosening their bonds and facilitating their dissociation [15]. Therefore, introducing inorganic materials into the junctions of BPMs increases the electric field strength, enhances water activity, and weakens water bonds, thereby promoting water-splitting [16]. However, excessive incorporation of metal catalysts can interfere with water polarization, and immobilized metal compounds may block ion-transfer channels within the membrane, resulting in increased membrane resistance. Thus, incorporating an appropriate amount of catalyst at the bipolar junction is essential [15,17].

Among various metal compounds, iron-based particles (e.g., Fe(OH)_3_ or Fe_3_O_4_) are known as representative metal catalysts that can enhance the water-splitting performance of BPMs [16,18]. Recently, considerable research has focused on developing iron-based water-splitting catalysts. For example, Wang et al. developed an Fe-MIL-101-NH_2_ catalyst with a metal–organic framework (MOF) structure that contains weak acid groups and metal centers [19]. In their study, the optimal amount of catalyst was determined through electrochemical analysis, and excellent water-splitting performance was demonstrated. The enhanced performance was attributed to the porous MOF structure as well as the presence of amino groups and iron ions. The fabricated catalyst exhibited a water-splitting voltage of approximately 4.2 V in a 2.0 M NaCl aqueous solution and maintained its initial water-splitting voltage for 6 h at a current density of 50 mA cm^−2^. Meanwhile, Cheng et al. enhanced water-splitting performance by introducing a KFe[Fe(CN)_6_] catalyst at the bipolar membrane interface [20]. They reported that the high catalytic activity of the developed catalyst was closely associated with its cubic complex structure and multiple catalytic sites. Furthermore, Ge et al. developed an Fe(III)@PEI catalyst in which iron ions are coordinated to the polymer matrix [21]. They found that the catalyst not only promoted the water-splitting reaction but also prevented catalyst leakage by stabilizing the iron ions as a metal complex through coordination interactions. The Fe(III)@PEI-incorporated BPM exhibited a voltage of 1.88 V at a current density of 320 mA cm^−2^, along with a significantly slower voltage increase rate (5.93 mV h^−1^) compared with the FeCl_3_-based BPM (32.64 mV h^−1^) at a constant current density of 60 mA cm^−2^. These results confirm that the metal–polymer coordination complex junction contributes to enhancing the durability of BPMs. In addition, Kim et al. fabricated a composite catalyst (Fe_2_O_3_@GO) by growing hematite (*α*-Fe_2_O_3_) nanoparticles (NPs) on two-dimensional graphene oxide (GO) nanosheets [22]. The partially dissociated bound water induced by the strong Lewis acidity of the Fe atoms in the Fe_2_O_3_@GO catalyst further compacted the “ice-like water” structure, weakening the O–H bonds of water molecules and thereby enhancing the water-splitting reaction rate. As a result, the Fe_2_O_3_@GO-incorporated BPM exhibited a significantly lower water-splitting potential (0.89 V) at a current density of 100 mA/cm^2^ compared with a commercial BPM (BP-1E, Astom Corp., Tokyo, Japan, 1.13 V) [20]. The literature reviewed above highlights the need to improve the stability of Fe-based catalysts, which are readily soluble in water within BPM systems. For example, iron hydroxide (Fe(OH)_3_) is widely used as a commercial membrane water-splitting catalyst because of its simple synthesis and excellent catalytic performance [23]. However, iron hydroxide exhibits higher solubility in water than iron oxide, and its solubility varies substantially with pH [24].

Therefore, in this study, composite NPs (Fe_3_O_4_-chitosan core–shell) fabricated by combining iron oxide and chitosan, which are suitable for DSWE, were introduced at the bipolar junction as a catalyst to develop a BPM with low water-splitting resistance, high mechanical strength, and enhanced catalytic stability. Magnetite (Fe_3_O_4_) is a magnetic material with an inverted spinel cubic structure and is insoluble in water. Recently, Fe_3_O_4_ NPs have attracted attention as catalyst supports due to their high loading capacity, excellent dispersibility, superior stability, and convenient recyclability [25]. However, iron oxide NPs are very small and have a high surface-to-volume ratio, which makes them prone to agglomeration and oxidation in air, leading to reduced stability. Therefore, surface modification is necessary to stabilize the NPs and prevent oxidation. In addition, surface modifiers can introduce functional groups that interact with other molecules [26]. Meanwhile, chitosan is a substance obtained through the deacetylation of chitin. Chitosan exhibits a high binding affinity for metal ions through chelation or ion exchange, which allows it to stably bind to metal oxide NPs such as Fe_3_O_4_ [25,27]. Furthermore, the hydroxyl and amine groups in chitosan can serve as functional sites that promote water dissociation. However, while chitosan is stable under neutral conditions, its physical stability in water decreases at pH values below 6.5. To address this limitation, chitosan can be cross-linked using a suitable agent, which helps maintain its stability in both acidic and alkaline environments [28]. Therefore, in this study, the physical stability of chitosan was enhanced by cross-linking it with glutaraldehyde (GA). In addition, a porous polyethylene (PE) substrate was used as a support to fabricate a base membrane, resulting in a thin BPM with excellent mechanical strength. Structural analysis and electrochemical characterization were performed for the fabricated AEL, CEL, and catalyst. The optimal catalyst loading was also determined through evaluation of water-splitting performance. Finally, the BPM prepared under optimal catalytic conditions was applied to a DSWE to verify its process performance. In this study, artificial seawater was used as the catholyte for the DSWE experiments, while a NaOH solution was employed as the anolyte to suppress the undesirable ClER.

## 2. Materials and Methods

### 2.1. Materials

The AEL, which constitutes the BPM, was fabricated by filling an ionomer into a porous PE support (thickness ≈ 23 μm, Hipore, Asahi Kasei E-materials, Tokyo, Japan). For the AEL ionomer, styrene (Sty) and 4-vinylbenzyl chloride (VBC) were used as monomers, divinylbenzene (DVB) as a cross-linker, benzoyl peroxide (BPO) as an initiator, and trimethylamine (TMA) solution for quaternization. To fabricate the CEL, polyether ether ketone (PEEK, 450 PF, *M_w_* = 39,200, Victrex, Lancashire, UK) was used as the base polymer, sulfuric acid as the sulfonation agent, and *N*,*N*-dimethylacetamide (DMAc) as the solvent. Iron (II, III) oxide (50–100 nm particle size), GA, acetic acid, oleic acid, and chitosan were used to prepare the iron oxide-based catalyst particles. For comparison, an iron hydroxide catalyst was prepared using iron (III) chloride and sodium hydroxide. The resulting catalyst powder was dispersed in 2-propanol (IPA) to produce the catalyst dispersion solution. The aqueous solutions used to evaluate BPM water-splitting characteristics and DSWE performance were prepared from sodium chloride, commercial sea salt, and sodium hydroxide. All reagents were purchased from Sigma-Aldrich (St. Louis, MO, USA) and used without further purification. Also, for benchmark comparison, BP-1E was selected as the commercial BPM.

### 2.2. AEL and CEL Preparation

**Figure 2:**
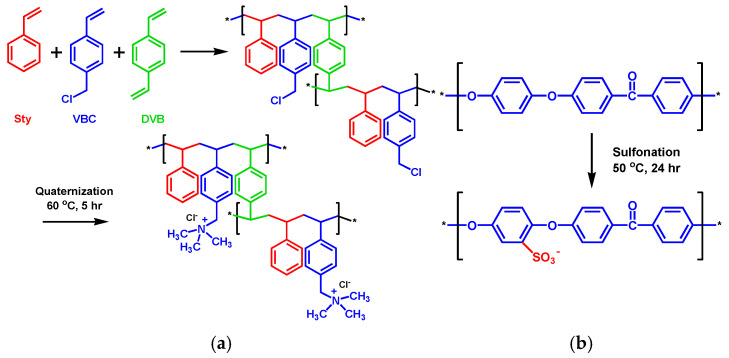
Fabrication procedures for (**a**) AEL and (**b**) CEL (The asterisk (*) in the structural formula indicates the connection point of the repeat unit).

To prepare the AEL, the porous PE support was first immersed in acetone for 1 h and then dried in an oven (OF-12GW, JEIO TECH, Daejeon, Republic of Korea) at 80 °C for 3 h. The monomers VBC and Sty were mixed at a 3:1 molar ratio, and the cross-linker DVB was added at 6 wt% of the monomer weight. The initiator BPO was then added at 2 wt% of the total monomer weight and stirred for 1 h. The porous support was immersed in the monomer mixture for 1 h to ensure sufficient penetration of the monomers into the pores. The monomer-filled support was then placed between two release films, laminated (PL-3500Plus, PAPER FRIEND by Hyundai Office, Daejeon, Republic of Korea), and polymerized in an oven at 80 °C for 3 h. After polymerization, the base membrane was immersed in a 1.0 M TMA aqueous solution and quaternized in an oven at 50 °C for 4 h. The resulting anion-exchange membrane (AEM) was washed several times with distilled water and then stored in a 0.5 M NaCl aqueous solution at room temperature [29]. The cation-exchange polymer for the CEL coating was prepared by sulfonating PEEK. First, PEEK powder was dissolved in sulfuric acid at a concentration of 10 wt% and stirred for 24 h under a nitrogen atmosphere at 50 °C. After the reaction was complete, the polymer solution was precipitated in distilled water and washed multiple times to remove residual sulfuric acid. The polymer was then dried in an oven at 80 °C for 24 h to yield sulfonated PEEK (SPEEK). The prepared SPEEK was dissolved in DMAc at a concentration of 20 wt% to produce the polymer solution for CEL fabrication [30]. Figure 2 illustrates the fabrication procedures of the AEL and CEL.

### 2.3. Fe(OH)_3_ and Fe_3_O_4_-Chitosan Core–Shell Catalysts Fabrication

First, to prepare the iron hydroxide catalyst, 100 mL of a 0.5 M NaOH aqueous solution was vigorously stirred, and 20 mL of a 0.1 M FeCl_3_ solution was slowly added dropwise. The reaction was continued until the red precipitate turned yellow. The resulting yellow iron hydroxide precipitate was filtered and dried in an oven at 80 °C for 1 h. Next, 0.025 mL of 0.5 M NaOH was added to 10 mL of a 50 *v*/*v*% IPA solution mixed with distilled water to adjust the pH to 11. Then, 0.1 wt% of the prepared iron hydroxide powder was added to this IPA/H_2_O/NaOH solution and sonicated for 30 min to obtain a well-dispersed catalyst solution.

**Figure 3:**
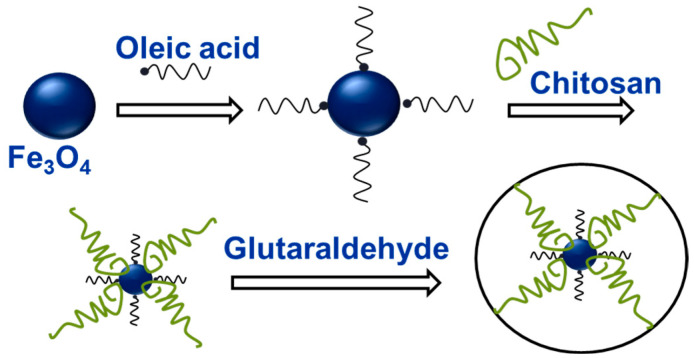
A schematic illustration for formation of Fe_3_O_4_–chitosan core–shell catalyst.

To prepare the Fe_3_O_4_–chitosan catalyst, 2.5 g of Fe_3_O_4_ particles were first added to 100 g of distilled water along with 3 mL of oleic acid, and the mixture was stirred at 75 °C for 30 min. The Fe_3_O_4_ particles were then separated, thoroughly washed, and dried in an oven at 80 °C for 24 h. Next, 0.5 g of chitosan was dissolved in 100 mL of a 2 wt% acetic acid solution, and 1.5 g of Fe_3_O_4_ particles were added. The mixture was sonicated for 20 min. Subsequently, 2 mL of GA solution was added, and the suspension was sonicated for 1 h at 40 °C. The resulting Fe_3_O_4_–chitosan core–shell particles were collected, thoroughly washed, and dried in an oven at 80 °C for 24 h. A total of 0.2 g of the dried catalyst particles was dispersed in 200 mL of a 2 wt% acetic acid solution and sonicated for 10 min. Subsequently, the particles were collected, washed with deionized water (DW), and dried in an oven at 80 °C to obtain a solid product. The resulting particles were then dispersed in a mixed dispersion medium of IPA and DW at a volume ratio of 1:1 to a final concentration of 0.1 wt% and used for subsequent experiments. As shown in Figure 3, the Fe_3_O_4_–chitosan particles exhibit a core–shell structure stabilized through cross-linking [31]. Because iron oxide particles tend to aggregate due to strong magnetic dipole–dipole interactions, oleic acid was used to prevent particle agglomeration, and cross-linking with GA was employed to improve the physical and chemical stability of the catalyst [31,32].

### 2.4. BPM Fabrication

Figure S1 schematically illustrates the BPM fabrication process. The previously prepared AEM was placed on a glass substrate and spray-coated with either the Fe(OH)_3_ or Fe_3_O_4_–chitosan catalyst solution. To identify the optimal catalyst loading, the catalyst solution was applied in 0.5 mL increments from 0.5 to 4.5 mL. In this procedure, the base membrane area was 18 cm^2^, and the glass substrate with the attached membrane was placed on a hot plate at 80 °C during the coating process. After catalyst coating, the SPEEK solution was cast onto the base membrane (catalyst-coated AEL) and dried in an oven at 80 °C for 6 h. The fabricated BPM was then stored in a 0.5 M NaCl aqueous solution until testing. To determine the actual catalyst loading, the AEM coated with a known amount of catalyst was immersed in concentrated sulfuric acid to dissolve the catalyst. The Fe concentration in the resulting solution was then measured using a DR/4000 spectrophotometer (Hach, Loveland, CO, USA), and the catalyst loading per unit area (μg cm^−2^) was calculated accordingly.

### 2.5. Characterizations of Catalysts and Membranes

The membrane electrical resistance (MER) of the IEMs was measured at room temperature in a 0.5 M NaCl aqueous solution using a lab-made two-point probe clip cell and a potentiostat/galvanostat (SP-150, Bio-Logic Science Instruments, Grenoble, France). The MER was calculated using Equation (1) [33]:

(1) $$\mathrm{MER}=\left(R_{1}-R_{2}\right)\times A\;\left[\Omega \;{\mathrm{cm}}^{2}\right]$$

where *R*_1_ is the resistance of the electrolyte and membrane (Ω), *R*_2_ is the resistance of the electrolyte (Ω), and *A* is the effective membrane area (cm^2^). The ionic conductivity (*σ*) was calculated using Equation (2) based on the measured electrical resistance:

(2) $$\sigma =\frac{L}{\mathrm{MER}}\;\left[{S\;\mathrm{cm}}^{-1}\right]$$

where *L* is the membrane thickness (cm). The water uptake (WU) was determined from the difference between the wet weight (*W_wet_*) and dry weight (*W_dry_*) of the membrane and calculated using Equation (3) [34]:

(3) $$\mathrm{WU}=\frac{W_{wet}-W_{dry}}{W_{dry}}\times 100\;\left[\%\right]$$

The ion-exchange capacity (IEC) of AEM was measured using the Mohr method. After the IEM reached equilibrium in a 0.5 M NaCl solution, the membrane surface was thoroughly rinsed with distilled water and then immersed in a 0.25 M Na_2_SO_4_ solution for at least 6 h to ensure complete exchange of Cl^−^ ions with SO_4_^2−^ ions inside the membrane. The concentration of Cl^−^ released into the solution was quantitatively determined by titration with a 0.01 M AgNO_3_ standard solution, using K_2_CrO_4_ as an indicator. The IEC of CEM was determined by acid–base titration. The membrane was first immersed in a 0.5 M HCl solution and then placed in a 0.5 M NaCl solution for more than 6 h to facilitate the exchange of Na^+^ ions with H^+^ ions in the membrane. The released H^+^ ions in the solution were titrated with a 0.01 N NaOH standard solution until the colorless solution turned pink. A 1 wt% phenolphthalein solution was used as the indicator. The IEC value was calculated using Equation (4) [34]:

(4) $$\mathrm{IEC}=\frac{C\cdot V_{s}}{W_{dry}}\;\left[\frac{\mathrm{meq}.}{g_{\mathrm{dry}\;\mathrm{memb}}}\right]$$

where *C* is the normality of the titrant (meq L^−1^), *V_s_* is the titrated solution volume (L), and *W_dry_* is the dry weight of the membrane (g). The transport number (TN, *t*^−^ or *t*^+^), which indicates the permselectivity of the IEM, was measured using the emf method with a lab-made two-compartment cell and calculated using Equations (5) and (6) [35].

(5) $$E_{m}=\frac{RT}{F}(1-2t^{-})\ln\frac{C_{L}}{C_{H}}$$

(6) $$t^{+}+t^{-}=1$$

In these equations, *E_m_* is the measured membrane potential, *R* is the gas constant, *T* is the absolute temperature, *F* is the Faraday constant, and *C_L_* and *C_H_* denote the NaCl concentrations, fixed at 1 mM and 5 mM, respectively. The membrane potential was recorded using a pair of Ag/AgCl electrodes connected to a digital multimeter (2010 Series, Keithley, Cleveland, OH, USA). The chemical structures of the fabricated ionomer and catalyst were analyzed using Fourier transform infrared spectroscopy (FT-IR, FT/IR-4700, Jasco, Hachioji, Japan). Field emission scanning electron microscopy (FE-SEM, MIRA LMH, TESCAN, Brno, Czech Republic) was used to observe the morphology of the pore-filled membrane employed as the BPM base layer, as well as the structures of the fabricated catalysts. In addition, the BPM samples were immersed in liquid nitrogen, fractured, and examined to obtain cross-sectional images for determining membrane thickness. To observe the core–shell structure of the Fe_3_O_4_–chitosan catalyst, transmission electron microscopy (TEM, JEM-F200, Jeol, Tokyo, Japan) was used. X-ray diffraction (XRD, MiniFlex 600, Rigaku, Tokyo, Japan) and X-ray photoelectron spectroscopy (XPS, K-Alpha, Thermo Fisher Scientific, Waltham, MA, USA) were performed to confirm the crystallinity and elemental composition of the prepared catalysts. The mechanical strength of the fabricated membranes was evaluated in the wet state using a universal testing machine (34SC-1, Instron, Norwood, MA, USA) in accordance with the ASTM D-882-79 standard [36].

### 2.6. Water-Splitting Performance Evaluation for BPMs

A two-compartment cell was used to evaluate the water-splitting characteristics of the BPMs. The effective cell area was 0.785 cm^2^, and each chamber was filled with 130 mL of a 0.5 M NaCl solution. Ag/AgCl electrodes were positioned at both ends, and a voltage of 4 V was applied for 10 min using a power supply (MK3003D, mkpower, Seoul, Republic of Korea). During the experiment, the pH change in the base compartment was monitored, and the water-splitting flux was subsequently calculated. *J*–*V* curves were also measured at room temperature using a two-compartment cell as shown in Figure S2. Both chambers were filled with 150 mL of a 0.5 M NaCl solution, and a pair of Ag/AgCl reference electrodes was positioned near the membrane. A potentiostat/galvanostat (SP-150, Bio-Logic Science Instruments, Grenoble, France) was connected to a pair of Pt-plated Ti (Pt@Ti) electrodes placed at both ends, and the *J*–*V* curve was recorded at a scan rate of 0.1 mA/s [23]. To evaluate catalyst stability, the BPM prepared with the optimal catalyst loading was immersed in distilled water at 60 °C and periodically sampled to measure the *J*–*V* curve. For the chronopotentiometry measurement, a two-compartment cell was filled with 150 mL of a 0.25 M Na_2_SO_4_ solution in each chamber. Ag/AgCl reference electrodes were positioned near the membrane, and Pt@Ti electrodes were installed at both ends. A constant current density of 38.2 mA cm^−2^ was applied for 12 h, and the time-dependent voltage response was recorded [22].

### 2.7. DSWE Performance Test

As shown in Figure S3, a lab-made two-compartment batch electrolyzer was used to evaluate the DSWE performance employing the BPM. Each chamber was filled with 120 mL of electrolyte solution, using 0.5 M NaOH as the anolyte and artificial seawater (50 mS cm^−1^) as the catholyte. At this stage, an alkaline solution free of Cl^−^ ions was used as the anolyte to prevent the undesirable ClER [8]. Pt@Ti mesh electrodes were used as both the cathode and anode, with an effective electrode area of 7.065 cm^2^. A 1 T-thick silicone gasket was used to maintain a fixed distance between the electrodes and the membrane. The electrodes were connected to a power supply, and a current density of 20 mA cm^−2^ was applied. The cell voltage was monitored periodically. In addition, to confirm the electrochemical stability of the DSWE system, the two-compartment cell shown in Figure S3 was connected in a three-electrode configuration (WE/RE1/CE) and operated under a constant current density of 20 mA cm^−2^ for 100 h to monitor changes in the cathode potential (*E_c_*) over time.

## 3. Results and Discussion

**Figure 4:**
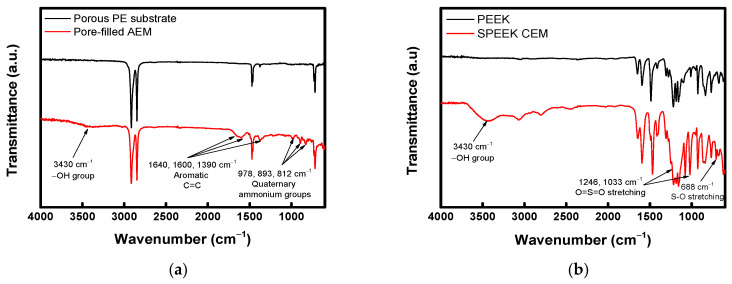
FT-IR spectra of (**a**) porous PE substrate and pore-filled AEM (AEL) and (**b**) PEEK and SPEEK CEM (CEL).

The FT-IR spectra of the fabricated ion-exchange layers (AEL and CEL) are presented in Figure 4. Figure 4a compares the spectra of the porous PE support and the pore-filled AEM. In the AEM spectrum, the broad peak at 3430 cm^−1^ corresponds to −OH groups originating from the hydration of ion-exchange sites [37]. The characteristic absorption peaks at 1640, 1600, and 1390 cm^−1^ indicate the presence of C=C bonds and aromatic rings [37,38,39]. Additionally, the peaks observed at 978, 893, and 812 cm^−1^ confirm the successful introduction of quaternary ammonium groups [40,41,42]. The spectra of the PEEK powder and the SPEEK CEM are also displayed in Figure 4b. Similarly to the AEM, a peak corresponding to –OH bonding was observed at 3430 cm^−1^. In addition, the introduction of sulfonic acid groups was confirmed by the absorption peaks at 1246, 1033, and 688 cm^−1^ [41].

The results of the basic characterization of the fabricated membranes are summarized in Table 1. The properties of the prepared AEM and CEM were compared with those of the commercial membranes, AMX and CMX (Astom Corp., Tokyo, Japan). The prepared membranes showed slightly higher IEC and WU values than the commercial membranes. In addition, they exhibited superior ion conductivity and lower electrical resistance, and their transport numbers were confirmed to be comparable to or higher than those of the commercial membranes. The ion-exchange layers that make up the BPM exhibit high ion conductivity, enabling rapid H^+^ and OH^−^ transport and resulting in high water-splitting efficiency. However, excessive WU can reduce mechanical stability and permselectivity, and may also lead to co-ion leakage from the BPM, ultimately lowering water-splitting efficiency. In contrast, the ion-exchange layers fabricated in this study demonstrated high permselectivity and an appropriate WU, indicating their suitability as components for BPMs.

Figure S4 presents the FT-IR spectra of chitosan and the synthesized Fe_3_O_4_–chitosan catalyst. The Fe_3_O_4_–chitosan spectrum shows the characteristic absorption peaks of chitosan, confirming its presence in the composite. In addition, the absorption peak at 592 cm^−1^ corresponds to the Fe–O vibration in the tetrahedral site of the spinel structure [43]. Meanwhile, crosslinking between the amino groups of chitosan and GA was confirmed, as the peak corresponding to the N–H vibration at 2360 cm^−1^ disappeared in the spectrum of Fe_3_O_4_–chitosan [44]. These results indicate that the Fe_3_O_4_ particles were successfully coupled with cross-linked chitosan.

**Figure 5:**
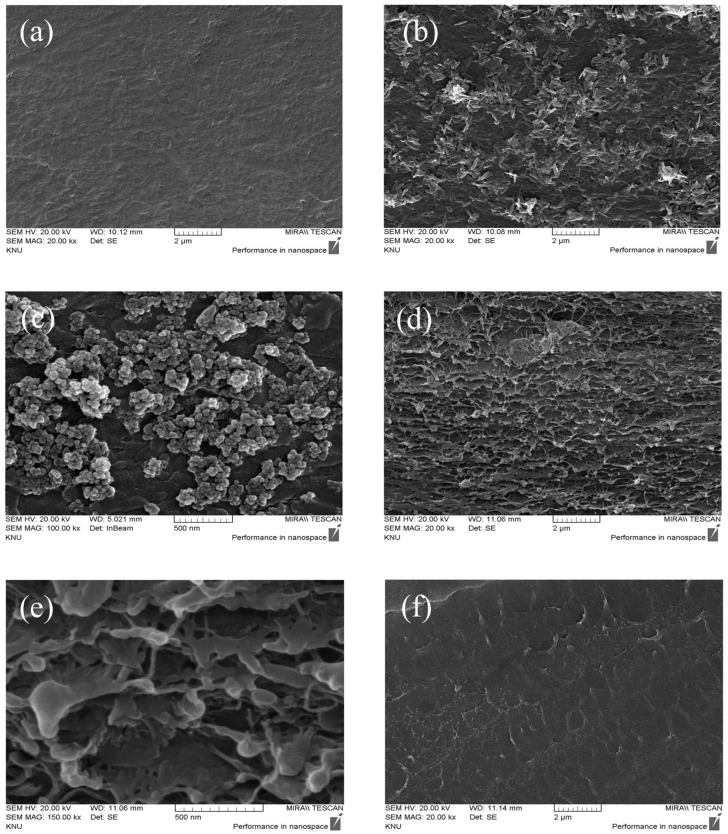

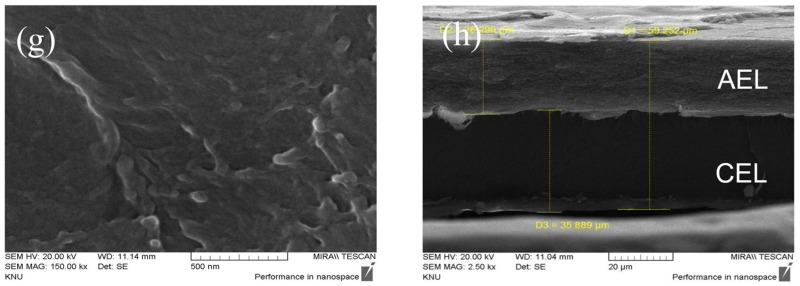
FE-SEM images of surface of (**a**) AEM, (**b**) Fe(OH)_3_-coated AEM, (**c**) Fe_3_O_4_–chitosan-coated AEM, and cross-section of (**d**) AEL (20.0 kX), (**e**) AEL (150.0 kX) (**f**) CEL (20 kX), (**g**) CEL (150 kX), and (**h**) BPM.

As shown in Figure 5, FE-SEM images were used to examine the morphology of the fabricated base membrane, catalysts, and BPM. Surface images of the AEM and the two types of spray-coated catalysts were obtained at a magnification of 20.0 kX and are presented in Figure 5a–c. The base membrane images confirm that the ionomer fully penetrated the pores of the porous support without observable defects. The catalyst images also show that Fe(OH)_3_ particles exhibited a plate-like morphology, whereas the Fe_3_O_4_–chitosan NPs were spherical. The spray-coated layers appeared relatively uniform across the membrane surface. Because variations in catalyst loading can locally increase interfacial resistance in the BPM and consequently reduce water-splitting efficiency, it is important to maintain a consistent coating thickness. Meanwhile, Figure 5d,e show the cross-sectional images of the AEL at magnifications of 20.0 and 150.0 kX, respectively. The images confirm that the porous support of the AEM is fully impregnated with the ionomer without any defects. Cross-sectional images of the CEL, obtained at the same magnifications, are presented in Figure 5f,g, and no specific issues were observed in its morphology. Figure 5h displays the cross-section of the BPM, where the AEL and CEL thicknesses are approximately 25 μm and 35 μm, respectively, resulting in a total BPM thickness of about 60 μm. Additionally, the catalyst layer was confirmed to be too thin to be detected in the FE-SEM image.

**Figure 6:**
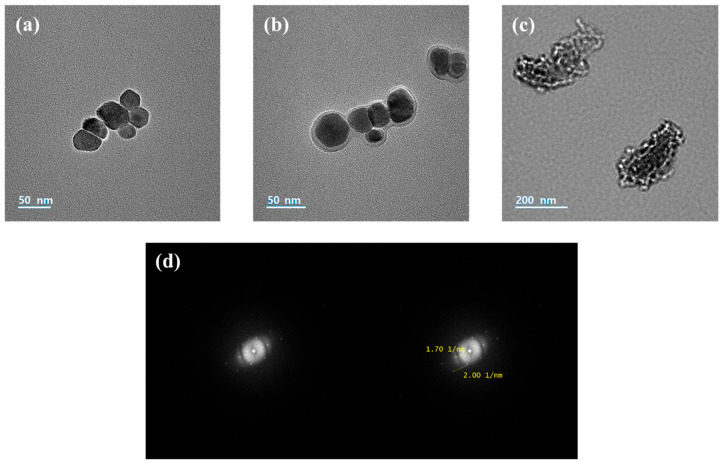
TEM images of (**a**) Fe_3_O_4_ NPs, (**b**) Fe_3_O_4_–chitosan NPs, (**c**) Fe(OH)_3_ NPs, and (**d**) SAED image of Fe_3_O_4_–chitosan NP.

To further investigate the morphology of the catalyst particles, TEM images were obtained, and the results are presented in Figure 6. As shown in Figure 6a–c, both the Fe_3_O_4_ and Fe_3_O_4_–chitosan core–shell particles exhibited an approximately spherical shape (approximately 50 nm in size), whereas the Fe(OH)_3_ particles appeared as amorphous plate-like structures. In addition, the TEM image of the Fe_3_O_4_–chitosan particles confirmed that the chitosan shell, approximately 2–5 nm thick, uniformly surrounded the Fe_3_O_4_ core. Figure 6d presents the selected area electron diffraction (SAED) pattern of the Fe_3_O_4_–chitosan core–shell particle. The pattern confirms that the synthesized Fe_3_O_4_–chitosan nanoparticles possess a cubic crystal structure with uniformly spaced diffraction rings. In addition, the radius of the diffraction rings in the SAED pattern was approximately 2.00 nm^−1^, corresponding to an interplanar spacing (*d*) of 1 nm.

**Figure 7:**
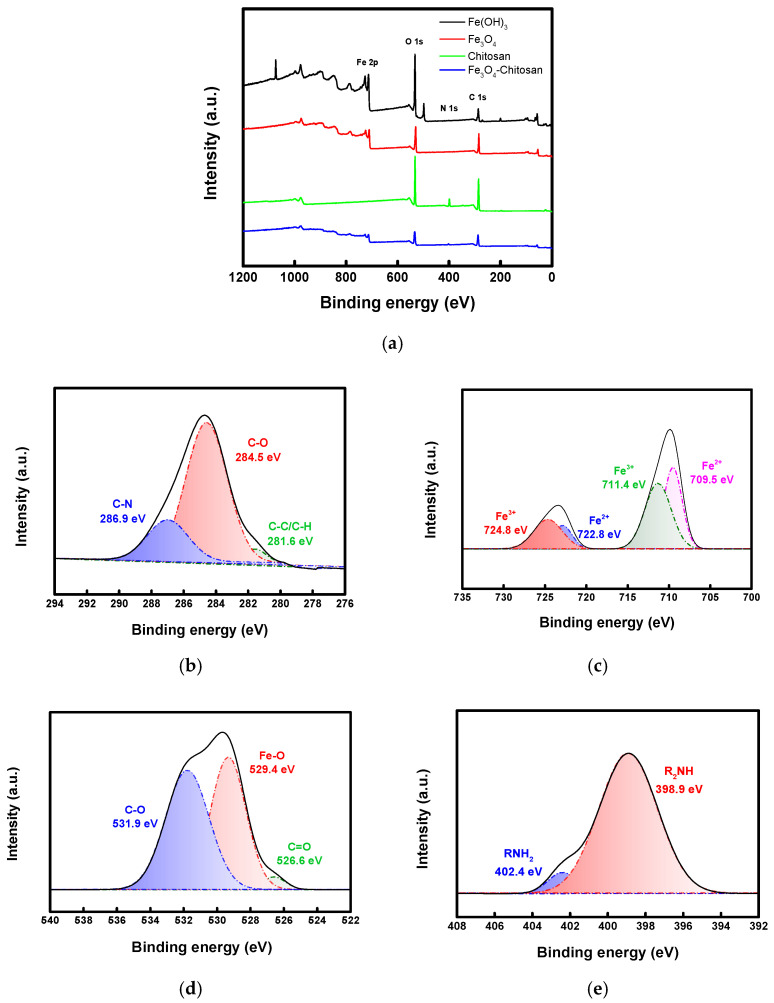
XPS spectra of (**a**) survey scan and fitting spectra with regard to (**b**) C ls, (**c**) Fe 2p, (**d**) O 1s, and (**e**) N 1s of chitosan and Fe_3_O_4_–chitosan NPs (Black line: fitted raw data).

Figure 7a displays the XPS spectra of the Fe_3_O_4_, chitosan, Fe_3_O_4_–chitosan, and Fe(OH)_3_ catalysts. In the spectrum of the Fe_3_O_4_–chitosan nanoparticles (NPs), the characteristic elemental peaks of both Fe_3_O_4_ and chitosan were detected. The spectra were deconvoluted, and the key results are shown in Figure 7b–e. Figure 7b displays the deconvolution of the C 1s peak, confirming the presence of C–O, C–N, C–C, and C–H bonds originating from chitosan. Furthermore, the Fe 2p spectrum in Figure 7c reveals the presence of Fe^2+^ in addition to Fe^3+^. This is because Fe_3_O_4_ inherently contains both Fe^3+^ and Fe^2+^ oxidation states [31,45]. Furthermore, the O 1s peak in Figure 7d shows not only the C–O bonding characteristic of chitosan but also a peak corresponding to Fe–O bonding at 529.4 eV. Meanwhile, as revealed in Figure 7e, the peak area corresponding to R_2_NH is much larger than that of RNH_2_, indicating that a substantial portion of the amino groups in chitosan were crosslinked through reaction with GA.

The mechanical properties of the fabricated and commercial BPMs in the wet state are compared in Table 2. Although the tensile strength of the fabricated BPM was slightly lower than that of the commercial membrane, it was still considered nearly equivalent. Meanwhile, the elongation at break was approximately 73% higher than that of the commercial membrane. Membranes fabricated using porous PE film supports tend to exhibit excellent tensile strength and high elongation at break [46,47]. Therefore, these excellent mechanical properties are attributed primarily to the characteristics of the support used for the membrane fabrication. Consequently, despite being much thinner than the commercial membrane, the fabricated BPM exhibited comparable mechanical strength and is considered suitable for practical applications.

**Figure 8:**
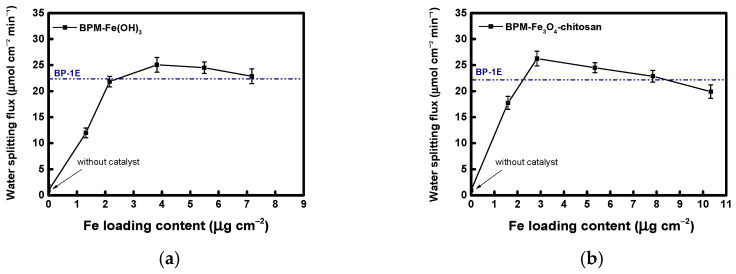
Water-splitting flux according to catalyst loading: (**a**) Fe(OH)_3_ and (**b**) Fe_3_O_4_–chitosan NPs.

The water-splitting performance of the fabricated BPMs at different catalyst loadings is shown in Figure 8. The water-splitting flux was calculated from the cumulative concentration of OH^−^ ions generated through the BPM over 10 min. For both catalysts (Fe(OH)_3_ and Fe_3_O_4_–chitosan), the water-splitting flux increased as catalyst loading increased, up to an optimal level. Beyond this point, the flux decreased. This decline is attributed to excessive catalyst at the bipolar junction, which increases ion-transfer resistance (for H^+^ and OH^−^ ions) and weakens the local electric field. As a result, the Fe(OH)_3_ catalyst exhibited optimal water-splitting performance at a loading of approximately 4 µg cm^−2^, while the Fe_3_O_4_–chitosan catalyst reached its maximum flux at a slightly lower loading of approximately 3 µg cm^−2^. Under these optimal conditions, both catalysts showed higher water-splitting fluxes than the commercial BP-1E membrane, with Fe_3_O_4_–chitosan outperforming Fe(OH)_3_. Overall, these results indicate that the Fe_3_O_4_–chitosan core–shell catalyst exhibits greater activity toward water dissociation than conventional Fe(OH)_3_.

**Figure 9:**
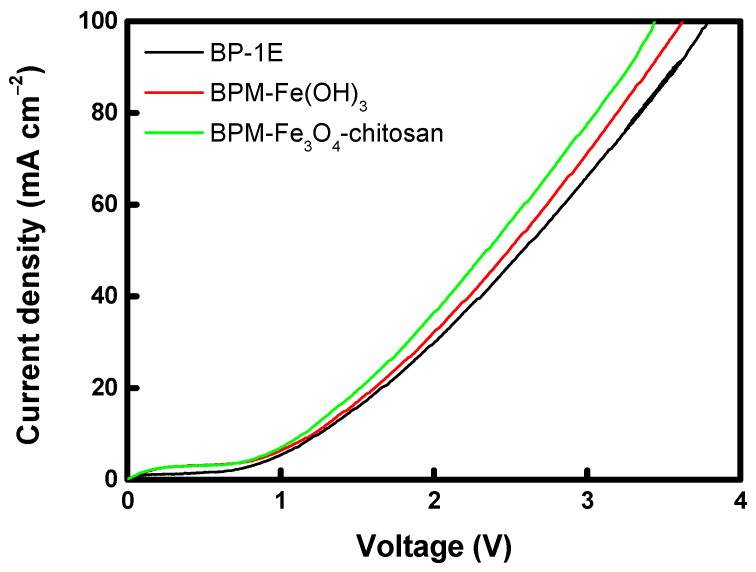
*J*–*V* curves of commercial and prepared BPMs.

To compare the water-splitting characteristics of BPMs prepared with different catalysts, *J*–*V* curves were measured as shown in Figure 9. As the applied voltage increases, the current initially rises due to ion migration within the membrane. Subsequently, a region of constant current (accompanied by a rapid increase in voltage) appears because all mobile ions in the BPM are depleted, preventing further charge transfer [48]. At this stage, when the remaining ions become insufficient to sustain the current, water-splitting occurs at the bipolar junction, where a strong electric field is established. In other words, the water-splitting reaction in the BPM begins once the limiting current density (LCD) is reached [49]. Beyond the LCD, the current begins to rise again once the applied voltage reaches the water-splitting region (approximately 0.83 V). This is because the H^+^ and OH^−^ ions generated by water dissociation at the bipolar junction act as new charge carriers. Therefore, a higher current at the same voltage indicates a faster water-splitting reaction in the BPM. The BPMs prepared with the optimal catalyst loading exhibited a steeper current increase than the commercial membrane, confirming their superior water-splitting performance. Meanwhile, the water-splitting onset voltage (*U_LCD_*) and the water-splitting voltage at 100 mA cm^−2^ (*U_100_*) were derived from the *J*–*V* curves and compared with those reported in previous studies, as summarized in Table S1 [16,21,50,51,52,53,54,55,56,57,58,59,60,61,62]. The comparison results confirm that the BPM–Fe_3_O_4_–chitosan developed in this study exhibits reasonable *U_LCD_* and *U_100_* values. However, direct comparison is challenging due to differences in evaluation environments and testing conditions for BPMs reported in the literature. Therefore, in this study, the performance of the developed BPMs was compared under identical experimental conditions with that of a commercial BPM (BP-1E), which is known to exhibit excellent performance.

Moreover, when comparing the two catalyst types, the BPM containing the Fe_3_O_4_–chitosan catalyst exhibited slightly higher water-splitting performance than that containing Fe(OH)_3_. This enhancement is attributed not only to the protonation–deprotonation reactions between the Fe-based transition metal catalyst and water molecules, but also to the amine and hydroxyl functional groups in chitosan, which further promote water dissociation. In addition, chitosan improves the hydrophilicity of metal oxide nanoparticles, thereby enhancing water-accessibility at the bipolar junction and contributing to improved water-splitting performance. When water molecules dissociate at the bipolar junction, continuous supply from the bulk solution is required. However, if the catalyst layer is relatively hydrophobic, this water supply can be hindered, ultimately reducing the water-splitting efficiency of the BPM [63].

Figure S5 also presents the *J*–*V* curves measured after immersing the samples in DW at 60 °C and removing them at predetermined intervals to evaluate catalyst stability. As the immersion time increased, the slope in the third region of the *J*–*V* curve and the current associated with water splitting gradually decreased, indicating a deterioration in BPM water-splitting performance. This decline is likely due to catalyst leaching from the bipolar junction under high-temperature conditions. In addition, the BPM–Fe(OH)_3_ exhibited a greater increase in resistance over time compared to the BPM–Fe_3_O_4_–chitosan, confirming that the Fe_3_O_4_–chitosan catalyst provides superior stability relative to Fe(OH)_3_.

**Figure 10:**
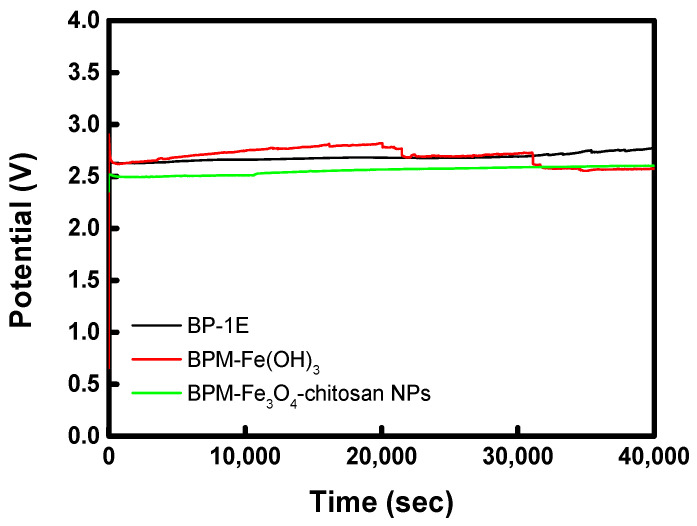
Electrochemical stability evaluated through chronopotentiometry at 38.2 mA cm^−2^ for 12 h.

Meanwhile, Figure 10 shows the chronopotentiometric curves of the commercial and prepared BPMs measured at a current density of 38.2 mA cm^−2^ for approximately 12 h. The BPM–Fe_3_O_4_–chitosan exhibited a lower potential than both the commercial membrane and BPM–Fe(OH)_3_. This is attributed to its higher water-splitting flux at the same current density, resulting in a lower water-splitting resistance. Both the BPM–Fe_3_O_4_–chitosan and BP-1E maintained stable potentials throughout the test, whereas the BPM–Fe(OH)_3_ showed slight potential fluctuations, likely due to catalyst dissolution.

**Figure 11:**
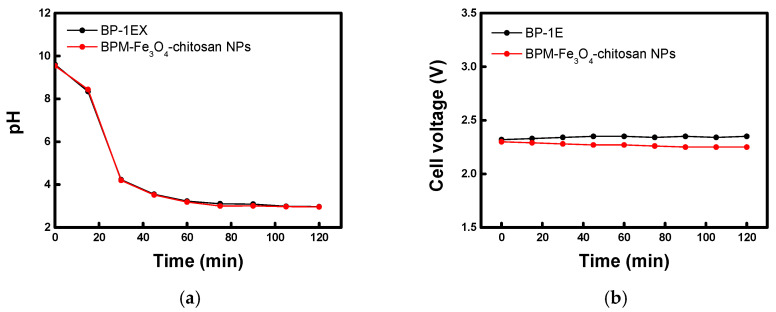
DSWE operation characteristics of commercial and Fe_3_O_4_–chitosan BPMs: (**a**) pH change in the catholyte and (**b**) cell voltage.

DSWE performance was evaluated using BP-1E and the prepared BPM with the optimal catalyst conditions, and the results are presented in Figure 11. Figure 11a shows the pH change in the catholyte over time when operating a BPM-based seawater electrolyzer (catholyte//BPM//0.5 M NaOH) at a constant current density of 20 mA cm^−2^. During operation, the catholyte steadily acidified, reaching and maintaining a pH of approximately 3 after 30 min. As shown in Figure 11b, the cell voltage also remained stable throughout the operation. The catholyte acidification occurs as follows: the high concentration of Mg^2+^ ions present in seawater reacts with the OH^−^ ions generated by the HER, thereby preventing alkalinization of the bulk catholyte. Simultaneously, the H^+^ ions generated by the BPM dissolve the hydroxide (Mg(OH)_2_) precipitates, and the remaining excess H^+^ ions acidify the seawater [4]. Therefore, if the water-splitting performance of the BPM is slower than the rate of Mg(OH)_2_ formation on the cathode, the catholyte pH cannot be lowered. These experimental results, therefore, indicate that the water-splitting performance of the fabricated membrane is sufficiently high to enable continuous and stable DSWE operation. Furthermore, as previously confirmed, the BPM–Fe_3_O_4_–chitosan membrane exhibited superior water-splitting performance compared to the commercial BP-1E membrane, resulting in relatively lower cell voltage.

Figure S6 shows photographs of the cathode chamber during DSWE operation as a function of membrane type and the presence or absence of a catalyst within the BPMs. As shown in Figure S6a, when an AEM was used, the pH of the cathode compartment increased, resulting in a significant increase in solution turbidity due to the formation of metal hydroxides. In contrast, as shown in Figure S6b,c, no solution turbidity was observed when BP-1E or the BPM–Fe_3_O_4_–chitosan was used. This indicates that the BPMs effectively acidify the catholyte, thereby suppressing the formation of inorganic precipitates. For comparison, when a BPM without a water-splitting catalyst was used (Figure S6d), high solution turbidity was observed. This behavior is attributed to the insufficient water-splitting rate of the BPM, which was unable to adequately suppress the formation of inorganic precipitates.

**Figure 12:**
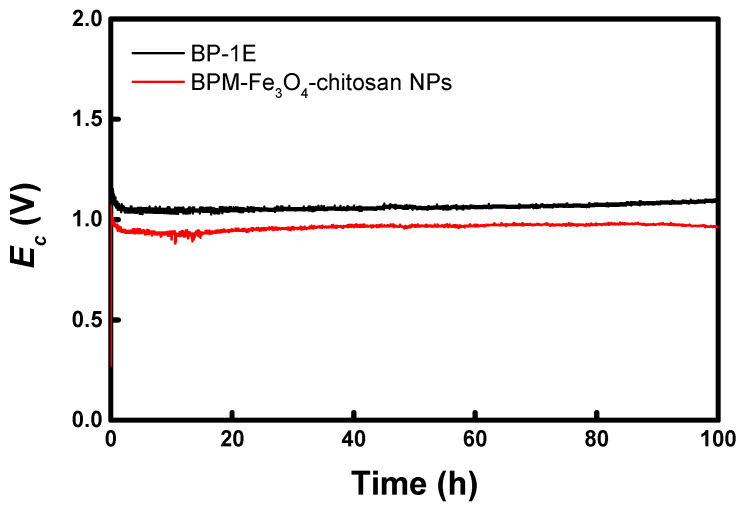
Chronopotentiometric curves for evaluating DSWE stability over 100 h (at 20 mA cm^−2^).

Furthermore, to more clearly assess the electrochemical stability of the DSWE system, chronopotentiometric curves were measured under constant-current operation at 20 mA cm^−2^ for 100 h, and the results are presented in Figure 12. The measured potential difference corresponds to the *E_c_*. A lower *E_c_* value indicates that water-splitting in the BPM enhances the transport of H^+^ ions to the cathode, thereby promoting the HER [8,22]. Concurrently, a high water-splitting flux of the BPM increases the concentration of free protons that do not directly participate in the HER, leading to a decrease in solution pH. This acidification suppresses the formation of inorganic precipitates and prevents an increase in electrode resistance [10]. The *E_c_* values of both the commercial membrane and the BPM–Fe_3_O_4_–chitosan membrane remained stable over 100 h, indicating excellent electrochemical stability. Meanwhile, the BPM–Fe_3_O_4_–chitosan membrane developed in this study exhibited a slightly lower *E_c_* than the commercial membrane, demonstrating its relatively superior water-splitting performance. In addition, the Fe concentration in the solution was analyzed using a DR/4000 Spectrophotometer during the DSWE experiment. This analysis was below the detection limit, confirming that no Fe leaching occurred under these conditions.

## 4. Conclusions

In this study, we developed a BPM with excellent water-splitting performance for DSWE applications. The anion-exchange base membrane (AEL) was prepared by filling a porous PE support with a styrene-based cross-linked ionomer, and the CEL was cast from SPEEK onto the base membrane. To enhance water-splitting performance, Fe(OH)_3_ and Fe_3_O_4_–chitosan catalysts were introduced into the bipolar junction via spray coating. The Fe_3_O_4_–chitosan catalyst was designed to improve dispersibility and hydrophilicity by using a chitosan shell around the Fe_3_O_4_ core. Functional groups in chitosan further accelerate water splitting, and cross-linking the amino groups with GA enhances nanoparticle stability. The resulting AEL and CEL exhibited high ion conductivity, permselectivity, and suitable water content, making them well-suited for BPM fabrication. The fabricated BPMs had a thickness of approximately 60 μm, and the porous PE support provided mechanical strength comparable to that of commercial membranes. Optimal catalyst loading was determined by evaluating water-splitting performance at different loading levels. The Fe(OH)_3_ catalyst showed optimal performance at ~4 μg cm^−2^, while the Fe_3_O_4_–chitosan catalyst was optimal at ~3 μg cm^−2^. Considering both water-splitting flux and optimal loading, the Fe_3_O_4_–chitosan catalyst exhibited higher water-splitting promotion activity than the conventional Fe(OH)_3_ catalyst. High-temperature dissolution tests and chronopotentiometry confirmed that the Fe_3_O_4_–chitosan core–shell catalyst exhibited superior stability compared to conventional Fe(OH)_3_ catalysts. The BPM fabricated under optimal catalytic conditions was applied to DSWE, effectively suppressing the formation of inorganic precipitates in the catholyte and maintaining stable acidification during operation. Note that, in this study, artificial seawater was used as the catholyte, while a NaOH solution was employed as the anolyte to prevent the undesired ClER during the evaluation of DSWE performance. The high-performance BPM developed in this study is expected to demonstrate superior functionality not only in DSWE but also in other electro-membrane processes, such as bipolar membrane electrodialysis (BMED) for acid–base production.

## Figures and Tables

**Table 1:** Basic properties of IEMs.

Membrane	WU (%)	IEC (meq g^−1^)	Thickness (μm)	Conductivity (mS cm^−1^)	MER (Ω cm^2^)	TN (-)
CMX (Astom corp.)	26.2 ± 1.40	1.60 ± 0.02	165.3 ± 0.47	5.862	2.82 ± 0.01	0.966
SPEEK CEM	30.4 ± 0.88	1.68 ± 0.03	35.0 ± 1.41	9.120	0.38 ± 0.04	0.977
AMX (Astom corp.)	21.1 ± 2.10	1.51 ± 0.05	134.7 ± 1.25	4.157	3.24 ± 0.03	0.970
PFAEM	28.5 ± 2.10	2.04 ± 0.02	30.2 ± 0.43	9.437	0.32 ± 0.01	0.972

**Table 2:** Mechanical properties of BPMs.

Membrane	BP-1E		BPM-Fe_3_O_4_-Chitosan	
	Tensile Strength (MPa)	Elongation at Break (%)	Tensile Strength (MPa)	Elongation at Break (%)
Wet state	30.9 ± 0.54	10.4 ± 0.34	28.0 ± 0.61	17.5 ± 0.98

## Data Availability

Data are contained within the article.

## Supplementary Materials

The following supporting information can be downloaded at: https://www.mdpi.com/article/10.3390/membranes16010023/s1, Figure S1: Schematic illustration of the BPM fabrication process; Figure S2: Configuration of measurement cell for *J*–*V* curve for BPMs (C=CEL/A=AEL); Figure S3: Schematic illustration of the experimental setup used for DSWE (C=CEL/A=AEL); Figure S4: FT-IR spectra of chitosan and Fe_3_O_4_–chitosan NPs; Figure S5: Time-course measurement of the *J*–*V* curves of BPMs containing (a) Fe(OH)_3_ and (b) Fe_3_O_4_–chitosan NPs (stored in DW at 60 °C); Figure S6: Photographs of the cathodic chamber after 30 min of operation using (a) AEM, (b) BP-1E, (c) BPM–Fe_3_O_4_, and (d) BPM-without catalyst; Table S1: Overview of the properties of BPMs reported in recent literature.

## References

[1] Maka A.O.M., Mehmood M. (2024). Green hydrogen energy production: Current status and potential. Clean Energy.

[2] Horri B.A., Ozcan H. (2024). Green hydrogen production by water electrolysis: Current status and challenges. Curr. Opin. Green Sustain..

[3] Kawaguchi K., Goto K., Konno A., Nohira T. (2023). Novel High-Temperature Alkaline Water Electrolysis Using Molten KOH–H_2_O System. J. Electrochem. Soc..

[4] Khan M.A., Al-Attas T., Roy S., Rahman M.M., Ghaffour N., Thangadurai V., Larter S., Hu J., Ajayan P.M., Kibria M.G. (2021). Seawater electrolysis for hydrogen production: Challenges and opportunities. Energy Environ. Sci..

[5] Liu J., Duan S., Shi H., Wang T., Yang X., Huang Y., Wu G., Li Q. (2022). Direct seawater electrolysis by suppressing chlorine evolution reaction. Angew. Chem. Int. Ed..

[6] Wu L., Xu Y., Wang Q., Zou X., Pan Z., Leung M.K.H., An L. (2025). Direct seawater electrolysis for green hydrogen production: Electrode designs, cell configurations, and system integrations. Energy Environ. Sci..

[7] Liu Y., Wang Y., Fornasiero P., Tian G., Strasser P., Yang X.-Y. (2024). Long-term durability of seawater electrolysis for hydrogen: From catalysts to systems. Angew. Chem. Int. Ed..

[8] Han J.-H., Jwa E., Lee H., Kim E.J., Nam J.-Y., Hwang K.S., Jeong N., Choi J., Kim H., Jeung Y.-C. (2022). Direct seawater electrolysis via synergistic acidification by inorganic precipitation and proton flux from bipolar membrane. Chem. Eng. J..

[9] Han J.-H. (2023). Long-Term Stability of Seawater Acidification and Its Effect on the Formation of Mg(OH)_2_ Films with a Hierarchical Porous Structure in Bipolar Membrane-Based Direct Seawater Electrolysis. J. Electrochem. Soc..

[10] Han J.-H., Bae J., Lim J., Jwa E., Nam J.-Y., Hwang K.S., Jeong N., Choi J., Kim H., Jeung Y.-C. (2023). Acidification-based direct electrolysis of treated wastewater for hydrogen production and water reuse. Heliyon.

[11] Hong E., Yang Z., Zeng H., Gao L., Yang C. (2024). Recent Development and Challenges of Bipolar Membranes for High Performance Water Electrolysis. ACS Mater. Lett..

[12] Zhao D., Xu J., Sun Y., Li M., Zhong G., Hu X., Sun J., Li X., Su H., Li M. (2022). Composition and Structure Progress of the Catalytic Interface Layer for Bipolar Membrane. Nanomaterials.

[13] Bui J.C., Lees E.W., Marin D.H., Stovall T.N., Chen L., Kusoglu A., Nielander A.C., Jaramillo T.F., Boettcher S.W., Bell A.T. (2024). Multi-scale physics of bipolar membranes in electrochemical processes. Nat. Chem. Eng..

[14] Simons R. (1993). High Performance Bipolar Membrane. U.S. Patent.

[15] Meng F., Cheng D., Lin M., Zuo K., Zhang Z.B. (2025). Review on crossover minimization and catalytic layer-promoted water dissociation in bipolar membranes. Appl. Energy.

[16] Parnämäe R., Mareev S., Nikonenko V., Melnikov S., Sheldeshov N., Zabolotskii V., Tedesco M. (2021). Bipolar membranes: A review on principles, latest developments, and applications. J. Membr. Sci..

[17] Mareev S.A., Evdochenko E., Wessling M., Kozaderova O.A., Niftaliev S.I., Pismenskaya N.D., Nikonenko V.V. (2020). A comprehensive mathematical model of water splitting in bipolar membranes: Impact of the spatial distribution of fixed charges and catalyst at bipolar junction. J. Membr. Sci..

[18] Adisasmito S., Khoiruddin K., Sutrisna P.D., Wenten I.G., Siagian U.W.R. (2024). Bipolar Membrane Seawater Splitting for Hydrogen Production: A Review. ACS Omega.

[19] Wang Q., Wu B., Jiang C., Wang Y., Xu T. (2017). Improving the water dissociation efficiency in a bipolar membrane with amino-functionalized MIL-101. J. Membr. Sci..

[20] Cheng G., Zhao Y., Li W., Zhang J., Wang X., Dong C. (2019). Performance enhancement of bipolar membranes modified by Fe complex catalyst. J. Membr. Sci..

[21] Ge Z., Shehzad M.A., Ge L., Zhu Y., Wang H., Li G., Zhang Z., Ge X., Wu L., Xu T. (2020). Beneficial Use of a Coordination Complex As the Junction Catalyst in a Bipolar Membrane. ACS Appl. Energy Mater..

[22] Kim B.S., Park S.C., Kim D.-H., Moon G.H., Oh J.G., Jang J., Kang M.-S., Yoon K.B., Kang Y.S. (2020). Bipolar membranes to promote formation of tight ice-like water for efficient and sustainable water splitting. Small.

[23] Hanada F., Hiraya K., Ohmura N., Tanaka S. (1997). Bipolar Membrane and Method for Its Production. European Patent.

[24] Cao X., Chen Y., Liang X., Li Y., Zhang W., Cai Z., Zhang T. (2023). Basic Research on Selective Extraction of Iron from Titanium Dioxide Waste Acid to Prepare Iron Phosphate Precursors. Separations.

[25] Hartati H., Subaer S., Hasri H., Wibawa T., Hasriana H. (2022). Microstructure and Antibacterial Properties of Chitosan-Fe_3_O_4_-AgNP Nanocomposite. Nanomaterials.

[26] Mahdavi M., Ahmad M., Haron M.J., Namvar F., Nadi B., AbRahman M.Z., Amin J. (2013). Synthesis, Surface Modification and Characterisation of Biocompatible Magnetic Iron Oxide Nanoparticles for Biomedical Applications. Molecules.

[27] Pounraj S., Somu P., Paul S. (2018). Chitosan and graphene oxide hybrid nanocomposite film doped with silver nanoparticles efficiently prevents biofouling. Appl. Surf. Sci..

[28] Wulandari I.O., Mardila V.T., Santjojo D.J.D.H., Sabarudin A. (2018). Preparation and Characterization of Chitosan-coated Fe_3_O_4_ Nanoparticles using Ex-Situ Co-Precipitation Method and Tripolyphosphate/Sulphate as Dual Crosslinkers. IOP Conf. Ser. Mater. Sci. Eng..

[29] Kim D.-H., Park J.-H., Seo S.-J., Park J.-S., Jung S.H., Kang Y.S., Choi J.-H., Kang M.-S. (2013). Development of thin anion-exchange pore-filled membranes for high diffusion dialysis performance. J. Membr. Sci..

[30] Kim D.-H., Kang M.-S. (2017). Cost-effective Bipolar Membranes for Efficient Electrochemical Water Dissociation. Chem. Lett..

[31] Qu J., Liu G., Wang Y., Hong R. (2010). Preparation of Fe_3_O_4_–chitosan nanoparticles used for hyperthermia. Adv. Powder Technol..

[32] Li G.-Y., Jiang Y.-R., Huang K.-L., Ding P., Chen J. (2008). Preparation and properties of magnetic Fe_3_O_4_–chitosan nanoparticles. J. Alloys Compd..

[33] Choi Y.-J., Park J.-M., Yeon K.-H., Moon S.-H. (2005). Electrochemical characterization of poly (vinyl alcohol)/formyl methyl pyridinium (PVA-FP) anion-exchange membranes. J. Membr. Sci..

[34] Chen H., Cong T.N., Yang W., Tan C., Li Y., Ding Y. (2009). Progress in electrical energy storage system: A critical review. Prog. Nat. Sci..

[35] Jeevananda T., Yeon K.-H., Moon S.-H. (2006). Synthesis and characterization of bipolar membrane using pyridine functionalized anion exchange layer. J. Membr. Sci..

[36] (1979). Standard Test Method for Tensile Properties of Thin Plastic Sheeting.

[37] Vengatesan S., Santhi S., Jeevanantham S., Sozhan G. (2015). Quaternized poly (styrene-co-vinylbenzyl chloride) anion exchange membranes for alkaline water electrolysers. J. Power Sources.

[38] Hermán V., Takacs H., Duclairoir F., Renault O., Tortaic J.H., Viala B. (2015). Core double–shell cobalt/graphene/polystyrene magnetic nanocomposites synthesized by in situ sonochemical polymerization. RSC Adv..

[39] Tang R., Zhang Y., Zhang Y., Yu Z. (2016). Synthesis and characterization of chitosan based dye containing quaternary ammonium group. Carbohydr. Polym..

[40] Lee S., Lee H., Yang T.-H., Bae B., Tran N.A.T., Cho Y., Jung N., Shin D. (2020). Quaternary ammonium-bearing perfluorinated polymers for anion exchange membrane applications. Membranes.

[41] Ghosh S., Dhole K., Tripathy M.K., Kumar R., Sharma R.S. (2015). FTIR spectroscopy in the characterization of the mixture of nuclear grade cation and anion exchange resins. J. Radioanal Nucl. Chem..

[42] Chen D., Hickner M.A. (2012). Degradation of imidazolium- and quaternary ammonium-functionalized poly(fluorenyl ether ketone sulfone) anion exchange membranes. ACS Appl. Mater. Interfaces.

[43] Pham X.N., Nguyen T.P., Pham T.N., Tran T.T.N., Tran T.V.T. (2016). Synthesis and characterization of chitosan-coated magnetite nanoparticles and their application in curcumin drug delivery. Adv. Nat. Sci. Nanosci. Nanotechnol..

[44] Li B., Shan C.-L., Zhou Q., Fang Y., Wang Y.-L., Xu F., Han L.-R., Ibrahim M., Guo L.-B., Xie G.-L. (2013). Synthesis, Characterization, and Antibacterial Activity of Cross-Linked Chitosan-Glutaraldehyde. Mar. Drugs.

[45] Pourmortazavi S.M., Sahebi H., Zandavar H., Mirsadeghi S. (2019). Fabrication of Fe_3_O_4_ nanoparticles coated by extracted shrimp peels chitosan as sustainable adsorbents for removal of chromium contaminates from wastewater: The design of experiment. Compos. B Eng..

[46] Zou W., Tang G., Peng K., Mo X., Hu H., Yang Z., Xu T., Ling R., Ma Y., Fang J. (2025). Robust and ultrathin pore-filling anion exchange membranes for water electrolysis. AIChE J..

[47] Song H.-B., Kim D.-H., Kang M.-S. (2022). Thin-Reinforced Anion-Exchange Membranes with High Ionic Contents for Electrochemical Energy Conversion Processes. Membranes.

[48] Zabolotskii V.I., Shel’deshov N.V., Gnusin N.P. (1988). Dissociation of water molecules in systems with ion-exchange membranes. Russ. Chem. Rev..

[49] Hsueh C.-L., Peng Y.-J., Wang C.-C., Chen C.-Y. (2003). Bipolar membrane prepared by grafting and plasma polymerization. J. Membr. Sci..

[50] Liu Y., Wang J. (2020). Performance enhancement of catalytic bipolar membrane based on polysulfone single base membrane for electrodialysis. J. Membr. Sci..

[51] Oener S.Z., Foster M.J., Boettcher S.W. (2020). Accelerating water dissociation in bipolar membranes and for electrocatalysis. Science.

[52] Al-Dhubhani E., Swart H., Borneman Z., Nijmeijer K., Tedesco M., Post J.W., Saakes M. (2021). Entanglement-enhanced water dissociation in bipolar membranes with 3D electrospun junction and polymeric catalyst. ACS Appl. Energy Mater..

[53] Kole S., Venugopalan G., Bhattacharya D., Zhang L., Cheng J., Pivovar B., Arges C.G. (2021). Bipolar membrane polarization behavior with systematically varied interfacial areas in the junction region. J. Mater. Chem. A.

[54] Shehzad M.S., Yasmin A., Ge X., Ge Z., Zhang K., Liang X., Zhang J., Li G., Xiao X., Jiang B. (2021). Shielded goethite catalyst that enables fast water dissociation in bipolar membranes. Nat. Commun..

[55] Chen L., Xu Q., Oener S.Z., Fabrizio K., Boettcher S.W. (2022). Design principles for water dissociation catalysts in high-performance bipolar membranes. Nat. Commun..

[56] Eswaraswamy B., Suhag A., Goel P., Mandal P., Chattopadhyay S. (2022). Potential of montmorillonite nanoclay as water dissociation catalyst at the interface of bipolar membrane. Sep. Purif. Technol..

[57] Xu Z., Wan L., Liao Y., Pang M., Xu Q., Wang P., Wang B. (2023). Continuous ammonia electrosynthesis using physically interlocked bipolar membrane at 1000 mA cm^−2^. Nat. Commun..

[58] Al-Dhubhani E., Tedesco M., de Vos W.M., Saakes M. (2023). Combined electrospinning–electrospraying for high-performance bipolar membranes with incorporated MCM-41 as water dissociation catalysts. ACS Appl. Mater. Interfaces.

[59] Bhowmick S., Qureshi M. (2023). Vanadium oxide nanosheet-infused functionalized polysulfone bipolar membrane for an efficient water dissociation reaction. ACS Appl. Mater. Interfaces.

[60] Yu W., Zhang Z., Luo F., Li X., Duan F., Xu Y., Liu Z., Liang X., Wang Y., Wu L. (2024). Tailoring high-performance bipolar membrane for durable pure water electrolysis. Nat. Commun..

[61] Zhang X., Li Y., Yuan Y., Wu C., Wang X., Liu Y., Han X. (2024). Efficient bipolar membranes with Ti_3_C_2_T_x_ nanosheets as advanced catalysts in the interfacial layers for water splitting. Chem. Eng. Res. Des..

[62] Luo F., Yu W., Li X., Liang X., Li W., Duan F., Wang Y., Ge X., Wu L., Xu T. (2025). Enhanced bipolar membranes for durable ampere-level water electrolysis. Energy Environ. Sci..

[63] Fu R.Q., Xu T.W., Yang W.H., Pan Z.X. (2004). Fundamental studies on the intermediate layer of a bipolar membrane Part II. Effect of bovine serum albumin (BSA) on water dissociation at the interface of a bipolar membrane. J. Colloid Interface Sci..

