# Supplementary material for: A Bipolar Membrane Containing Core–Shell Structured Fe3O4-Chitosan Nanoparticles for Direct Seawater Electrolysis
Source: Membranes (Basel). 2026 Jan 2;16(1):23. doi: 10.3390/membranes16010023 (PMC12843638; doi:10.3390/membranes16010023)
Supplement: Supplementary file 1 [file membranes-16-00023-s001.zip › membranes-4027253-supplementary.pdf]

Supplementary materials

A bipolar membrane containing core-shell structured Fe_3O_4-chitosan nanoparticles for direct seawater electrolysis

Hyeon-Bee Song, Eun-Hye Jang and Moon-Sung Kang*

Department of Green Chemical Engineering, Sangmyung University, Cheonan-si, Chungcheongnam-do 31066, Republic of Korea

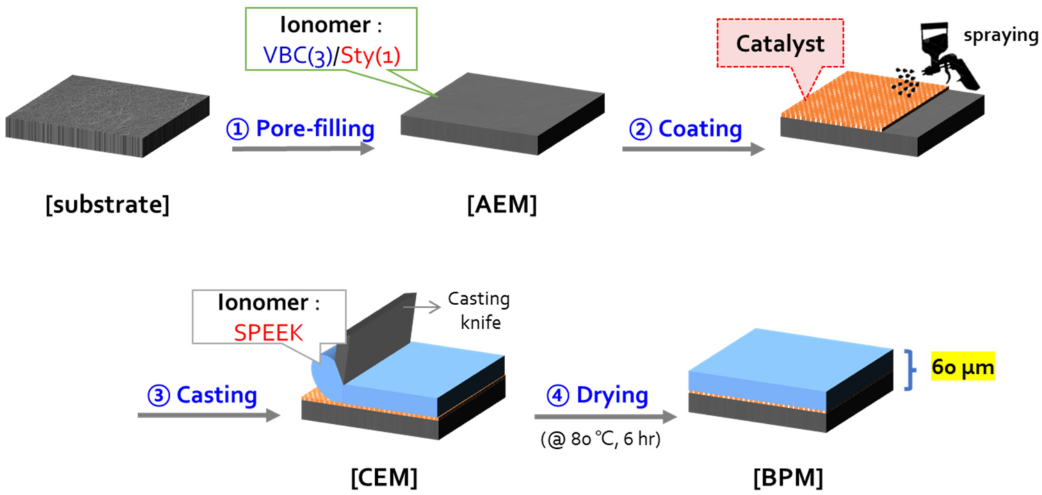

Figure S1. Schematic illustration of the BPM fabrication process.

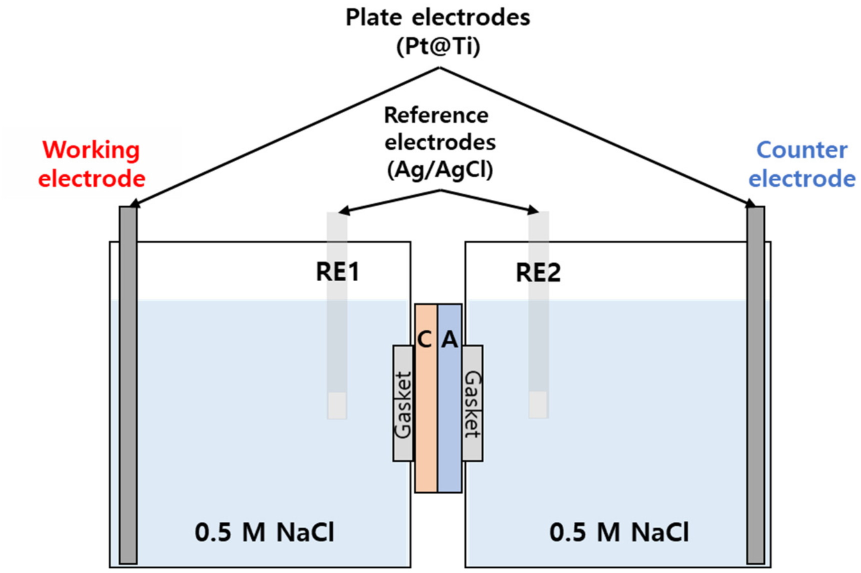

Figure S2. Configuration of measurement cell for *J*–*V* curve for BPMs (C=CEL/A=AEL).

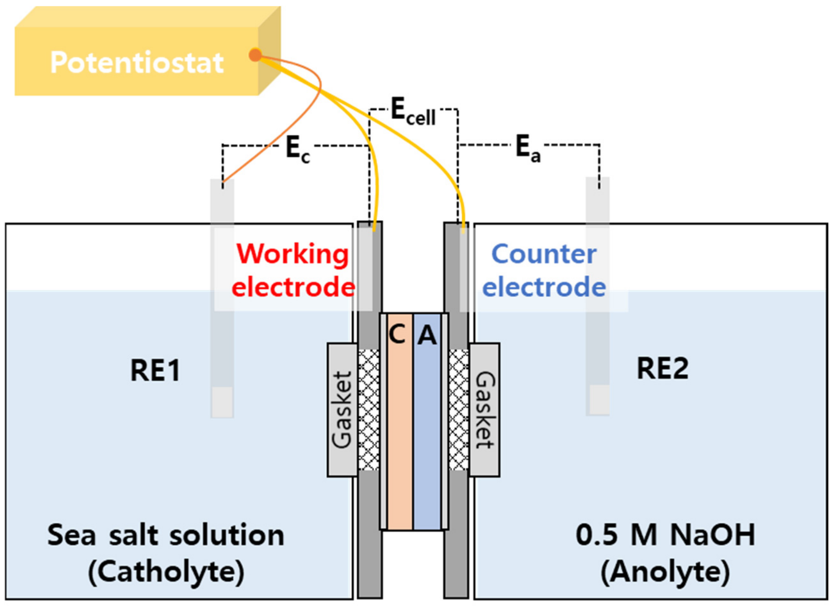

Figure S3. Schematic illustration of the experimental setup used for DSWE (C=CEL/A=AEL).

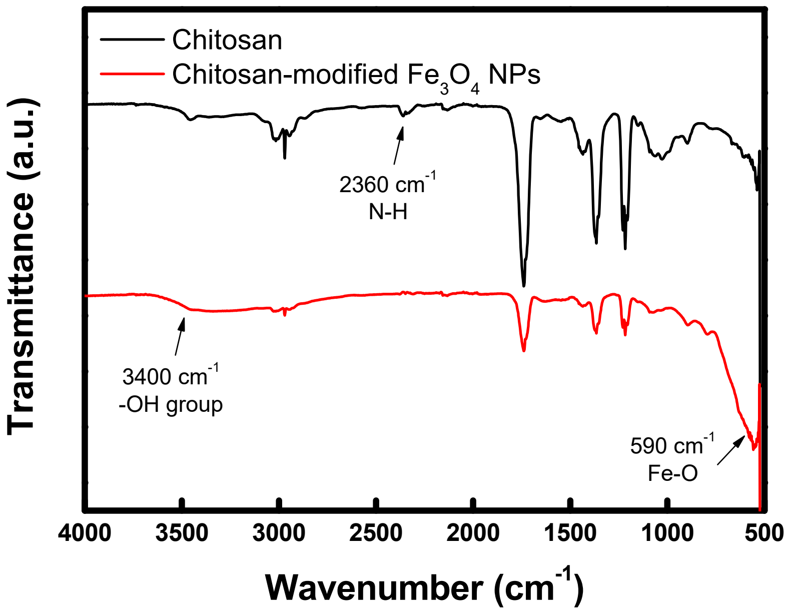

Figure S4. FT-IR spectra of chitosan and Fe_3O_4–chitosan NPs.

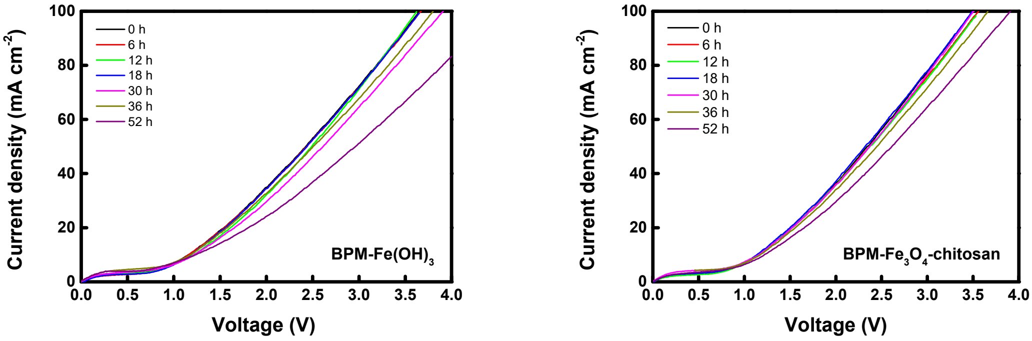

(a) (b)

Figure S5. Time-course measurement of the *J*-*V* curves of BPMs containing (a) $Fe(OH)_3$ and (b) Fe_3O_4–chitosan NPs (stored in DW at 60 ºC)

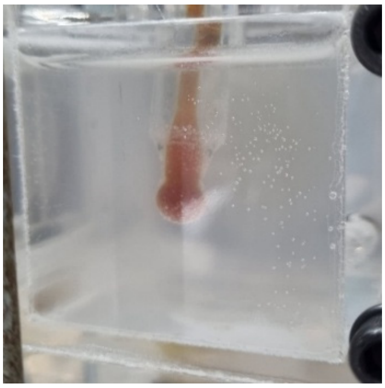

(a)

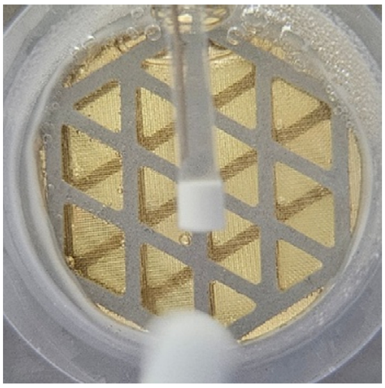

(b)

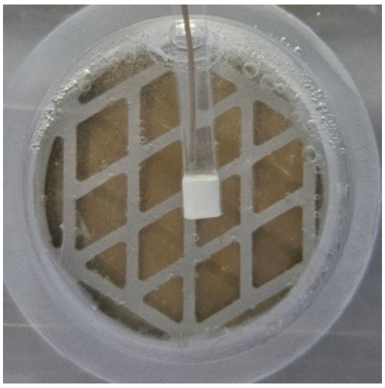

(c)

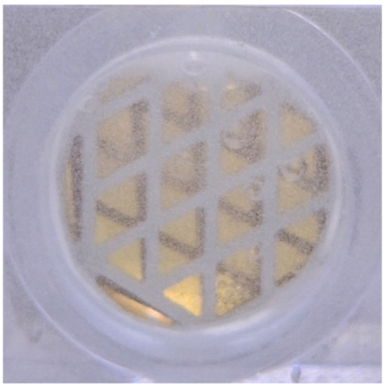

(d)

Figure S6. Photographs of the cathodic chamber after 30 min of operation using (a) AEM, (b) BP-1E, (c) BPM–Fe_3O_4, and (d) BPM-without catalyst.

Table S1. Overview of the properties of BPMs reported in recent literature.

Year	Membrane	CEL	AEL	Interfacial layer (catalyst)	U_{LCD} (V) (Test condition)	U_{100} (V) (Test condition)	Ref.
-	BP-1E (Neosepta)	Neosepta CM-1	Polysulfone	Iron (II, III), ruthenium (III), tin (II, IV); junction is roughened with sand paper	0.8 (0.5 M NaCl)	1.2 (1 N NaOH/HCl @ 10 A/dm^2 and 30°C)	[16]
-	FBM (Fumasep)	Crosslinked poly-ether ether ketone	- (Quaternary ammonium (bicyclic amines))	Insoluble polyelectrolyte complex with tertiary ammonium groups (poly acrylic acid/poly vinylpyridine salt complex)	0.84 (0.5 M NaCl)	1.39 (0.5 M NaCl)	[16]
2020	B-GO/PVA2	Sulfonated polysulfone (SPSF)	Quaternized polysulfone (QPSF)	GO+PVA	~2.10 (0.1 M NaCl)	~2.5 (0.1 M NaCl)	[50]
2020	B-Fe(Ⅲ)@PEI	Hydrolyzed polyacrylonitrile (HPAN)	Quaternized poly(phenylene oxide) (QPPO)	Fe(III)@PEI	1.88 (0.5 M NaCl)	1.88 (0.5 M NaCl)	[21]
2020	NiO-IrO$_2$ BPM	Nafion-212	Sustainion	NiO-IrO$_2$ bilayer	~0.75 (1.0 M HCl/NaOH)	N.A.	[51]
2021	BPM-E3	Sulfonated poly(ether ether ketone) (SPEEK)	FAA-3 (Fumasep)	Poly(4-vinylpyrrolidone) (P4VP)	<0.83 (0.5–2.0 M NaCl)	~1.00 (0.5 M NaCl)	[52]
2021	NIA-2.28	Sulfonated poly(ether ether ketone) (SPEEK)	Quaternized polysulfone (QPSF)	Al(OH)$_3$	~1.00 (1.0 M KNO$_3$)	~3.00 (1.0 M KNO$_3$ @ 50 mA/cm^2)	[53]
2021	SCBM	Nafion-212	Poly(phenylene oxide) (QPPO)	Goethite Fe^{+3}O(OH) at pre-PANI shield	0.8 (0.5 M NaCl)	1.1 (0.5 M NaCl)	[54]
2022	P25-TiO$_2$ catalyzed BPMs	Nafion™ 212	PiperION-A40-HCO$_3$ (TP-85, 40μm, Versogen)	P25-TiO$_2$	1.5	2.05 (0.5 M Na$_2$SO$_4$ @ 500 mA/cm^2)	[55]
2022	SBM-NC1.0	Sulfonated polyether-ether ketone (SPEEK)	Quaternized polysulfone	Montmorillonite (MMT) nanoclay + PVP	0.73 (0.5 M NaCl)	1.10 (0.5 M NaCl)	[56]
2023	MBM	Perfluorinated sulfonic acid	Quaternary ammonia poly (N-methyl-piperidine-co-p-terphenyl) (QPPT)	SnO$_2$	<0.8	1.13 (0.5 M Na$_2$SO$_4$ @ 1000 mA/cm^2)	[57]
2023	BPM-1h	Sulfonated poly(ether ether ketone) (SPEEK)	FAA-3	MCM-41 (zeolitic-like framework of amorphous silica)	0.6 (0.5 M NaCl)	1.0 (0.5 M NaCl)	[58]
2023	BPM_VO-ns	Sulfonated polysulfone (SPSF)	Quaternized polysulfone (QPSF)	(2D) V$_2$O$_5$ with PVA	1.11 (1 M NaCl)	3.6 (1 M NaCl)	[59]
2024	TPSn-BM	Sulfonated thioacetate group-tethered precursor	Quaternized thioacetate group-tethered precursor	SnO$_2$	~0.5 (0.5 M Na$_2$SO$_4$)	0.71 (0.5 M Na$_2$SO$_4$)	[60]

		polymer (p-TPSA)	polymer (p-TPQA)				
2024	BPM-PANI-$Ti_3C_2T_x$ -0.5%	Sulfonated poly(phenylene oxide) (SPPO)	Quaternized poly(phenylene oxide) (QPPO)	PANI–$Ti_3C_2T_x$	N.A.	7.0 (0.3 M NaCl @ 80 mA/cm^2)	[61]
2025	GO-NCA@BPM	Sulfonated poly (biphenyl indole) (SPBI)	Poly (meta/para-terphenylene-methyl piperidinium) (MTCP-50)	4-Tertiary amine calix[4]arene-modified graphene oxide (GO-NCA)	~0.5 (1 M KOH and 0.5 M H_2SO_4)	0.7 (1 M KOH and 0.5 M H_2SO_4)	[62]
2025	BPM-Fe_3O_4-chitosan	Sulfonated poly(ether ether ketone) (SPEEK)	Styrene (Sty) and 4-vinylbenzyl chloride (VBC)	Core-shell structured Fe_3O_4-chitosan nanoparticles	0.8 (0.5 M NaCl)	3.4 (0.5 M NaCl)	This work
